# Genome-wide association study and a post replication analysis revealed a promising genomic region and candidate genes for chicken eggshell blueness

**DOI:** 10.1371/journal.pone.0209181

**Published:** 2019-01-23

**Authors:** Hesham Y. A. Darwish, Seyed Benyamin Dalirsefat, Xianggui Dong, Guoying Hua, Jianfei Chen, Yuanyuan Zhang, Jianxiong Li, Jiansheng Xu, Junying Li, Xuemei Deng, Changxin Wu

**Affiliations:** 1 National Engineering Laboratory for Animal Breeding and Key Laboratory of Animal Genetics, Breeding, and Reproduction of the Ministry of Agriculture, China Agricultural University, Beijing, China; 2 Animal Production Research Institute, Agricultural Research Center, Ministry of Agriculture and Land Reclamation, Giza, Egypt; 3 Department of Animal Science, Faculty of Agricultural Sciences, University of Guilan, Rasht, Guilan, Iran; 4 Jiangxi Donghua Livestock & Poultry Breeding Co. Ltd., Jiangxi, China; Institute of Genetics and Developmental Biology Chinese Academy of Sciences, CHINA

## Abstract

The eggshell blueness is an interesting object for chicken genetic studies and blue-shelled chicken industry, especially after the discovery of the causative mutation of chicken blue eggshell. In the present study, genome wide association study (GWAS) was conducted in Chinese Dongxiang blue-shelled chicken underlying four traits of blue eggshell pigments: quantity of biliverdin (QB), quantity of protoporphyrin (QP), quantity of total pigment (QT), and color density trait (CD). A total of 139 individuals were randomly collected for GWAS. We detected two SNPs in genome-wise significance and 35 in suggestive significance, 24 out of the 37 SNP were located either within intron/exon or near 15 genes in a range of ~1.17 Mb on GGA21. For further confirmation of the identified SNP loci by GWAS, the follow-up replication studies were performed in two populations. A total of 146 individuals of the second generation derived from the former GWAS population, as well as 280 individuals from an alternative independent population were employed for genotyping by MALDI-TOF MS in a genotype-phenotype association study. Eighteen SNPs evenly distributed on the GGA21 significant region were successfully genotyped in the two populations, of which 4 and 6 SNP loci were shown significantly associated with QB, QT and QP in the two repeat populations, respectively. Further, the SNPs were narrowed down to a region of ~ 653.819 Kb on GGA21 that harbors five candidate genes: *AJAP1*, *TNFRSF9*, *C1ORF174*, *CAMTA1*, and *CEP104*. Shell gland of chickens laying dark and light blue eggshell was chosen for detection of mRNA expression of the five candidate genes. The results showed differential expression levels of these genes in the two groups. The specific function of these genes has not yet been defined clearly in chickens and further in-depth studies are needed to explore the new functional role in chicken eggshell blueness.

## Introduction

Eggshell color is considered one of the essential aspects of egg quality in many countries. Although there is little or no direct evidence to the relationship between eggshell color and the nutritional content of the egg, consumers tend to purchase eggs with particular colors [1]. Research on eggshell color intensity revealed that the uniformity of eggshell color is of things that are taken into account for consumers in the market [2]. Eggshell color results from an accumulation of pigments on the eggshell [3]. Basically, the blue eggs contain biliverdin-IX, zinc biliverdin chelate, and protoporphyrin-IX in the eggshells. Biliverdin refers to a type of bile pigment which in mammals, is subsequently converted to bilirubin by biliverdin reductase; however, in birds it is a major form of excretion due to the low effectiveness of biliverdin reductase [4, 5]. Lang and Wells [6] found that biliverdin-IX is a derivative of heme, which results from heme oxygenase-1 (HO-1) activity in the porphyrin pathway and plays an important role in the biochemistry of all living systems. The distribution of eggshell pigments on the two types of eggs (blue and brown eggshell) have been studied by Zhao et al. [7] and Wang et al. [8], they found that the eggshell from blue-shelled chickens contains a remarkable greater level of biliverdin and a lower level of protoporphyrin than the eggshell from brown-shelled chickens. In 1933, Punnett [9] firstly reported that blue or green shell appearance is determined by a single genetic factor and an autosomal dominant mutation, traditionally denoted as oocyan (O) and acts as normal Mendelian inheritance. Recent studies demonstrated that the blue eggshell phenotype in chickens is caused by an endogenous avian retroviral (EAV-HP) insertion in the 5' flanking region of the *SLCO1B3* gene. This retrovirus was shown to be associated with the over-expression of the *SLCO1B3* gene, proposing it to be the causative mutation of the oocyan phenotype in blue-shelled chickens [10, 11].

In China, Dongxiang chickens have been selected for blue eggshell and the population was fixed on the dominant homozygous genotype of *SLCO1B3* gene. Despite the fact that all of the hens lay blue-shelled eggs, the color intensity of the eggs varies from dark to light blue. Therefore, in the present study, a genome-wide association study on eggshell blueness was performed for the first time using a high-density 600K SNP array followed by two different replication association studies. We believe that identification of genes associated with the variations of blue eggshell color may provide a new insight into the genetic basis of these traits and benefit for designing an efficient selection strategy in laying hens breeding programs to produce eggs with uniform or desired colors.

## Materials and methods

### Ethics statement

All experimental procedures and animal use were approved by The Beijing Municipal Committee of Animal Management and The Ethics Committee of the China Agricultural University.

### Samples and phenotype measurements

For GWA study, blood and egg samples of 139 individuals aged 42 wk of blue eggshell resource population with dominant homozygous genotype of *SLCO1B3* gene were collected from China Agricultural University Research Farm. All birds were kept in single cages in one poultry house. Three eggs were collected from each hen on three consecutive days and analyzed while fresh. Eggshell color was measured using chemical and physical methods. In accordance with the chemical method, the experimental procedure to measure eggshell color traits, including quantity of biliverdin (QB), quantity of protoporphyrin (QP), quantity of total pigments biliverdin and protoporphyrin (QT) was followed as described by Wang et al. [8]. The average of 3 d was taken as phenotypic value of each trait for every hen. The quantities of biliverdin and protoporphyrin in mol/g were measured for each egg. In the physical method, eggshell color reflectance was measured *in situ* using an eggshell color analyzer (Model QCR, UK) following the instruction. To minimize instrument error, dark and white standard reflectance calibration measures were taken regularly during the sampling. The color reflectance value is based on 100% scale and represents the brightness of eggshell surface, whereas the darkness of eggshell is desired for density of eggshell pigment. Therefore, we calculated 100-reflectance value as color density (CD) representing the mixed concentration of biliverdin and protoporphyrin. All phenotypic data were tested for normality, and any abnormal values highly deviating from normal distribution were deleted.

### Genotyping and quality control (QC)

Two ml blood samples were collected from brachial veins of chickens by venipuncture. The blood samples obtained by venipuncture were immediately injected into 5 ml tubes containing K3-ethylenediaminetetraacetic acid anticoagulant and stored at -20 pending the extraction of DNA or any other hematological parameters measurement. Genomic DNA was extracted using TIANamp Blood DNA Kit (Tiangen, China).

The DNA concentration and its purity were examined using spectrophotometric analysis. A total of 139 individuals were quantified for DNA concentrations and genotyped using the 600 K Affymetrix Axiom Chicken Genotyping Array (Affymetrix, Inc. Santa Clara, CA, USA). Genotyping data were analyzed through the software package PLINK v1.09 [12]. Quality control of the genotype data was conducted at both the individual level and the SNP level [13]. At the individual level, five samples were excluded from each phenotype data set with a quality control criterion of missing rate per individual < 95%. At the SNP level, 5,325 SNPs with call rates < 0.95, 201,806 SNPs with minor allele frequencies < 0.1, 25,737 SNPs with P values from Hardy-Weinberg equilibrium test < 0.000001 were excluded from the analysis. After QC there were 134 subjects and 348,093 SNPs in the analysis data set. These filters were selected in accordance with procedures described by Anderson et al. [13] to minimize the influence of genotype-calling artifacts in a GWA study. Typically, a MAF threshold of 1–2% is applied, but studies with small sample sizes may require a higher threshold [13]. The final SNP set included 348,093 valid SNPs with average intervals of ~2.7 Kb, distributed across 28 autosomes (GGA1 to 28), two linkage groups (LGE22 and LGE64) and one unassigned group for genome-wide association analysis. All SNPs on sex chromosomes were discarded in QC process. The marker information on each chromosome before and after quality control is summarized in S1 Table.

### Linkage disequilibrium analysis

Pairwise linkage disequilibrium (LD) measured by r^2^ threshold for the population was calculated for each chromosome using PLINK (v1.09) software and the population structure was assessed using MDS analysis available from the same software. As suggested by Wang et al. [14], all autosomal SNPs were pruned using the “indep-pairwise” option, with a window size of 25 SNPs, a step of 5 SNPs, and r^2^ threshold of 0.2, resulting in 30,885 independent SNP markers.

### Stratification analysis

Pairwise identity-by-state (IBS) distances were calculated between all individuals using the 30,885 independent SNP markers. Two dimension reduction routines provided by PLINK 1.09 were performed to assess population structure: the “pca” for principal components analysis (PCA) based on the variance-standardized relationship matrix [15, 16], and “mds-plot” for multidimensional scaling (MDS) based on raw Hamming distances [17]. Top principal components were used as covariates in association analysis regressions to help for the correction of population stratification, while MDS coordinates were used for visualizing genetic distances. Plotting the component1 values against component2 was used to identify clustering of samples using standard classical (metric) multidimensional scaling. The quantiles of the observed P values for the GWAS experiment were compared with the quantiles of the standard normal distribution using a quantile-quantile plot (Q-Q plot) in the R-project software [18] to determine if there was an excess of extremely high observed values.

### Genome-wide association analyses

Genome wide association analyses were carried out in PLINK software (v1.09). Because pedigree of most individuals of the population under study was unknown, association tests were performed based on least squares (LS) regression test with the top 20 principal components as covariates for unrelated individuals and then Bonferroni correction was utilized to adjust multiple testing. The genomic inflation factor [19] was calculated for GWA analysis of each trait. Association was defined based on Bonferroni corrected P value thresholds at three levels of significance: chromosome-wise significance or suggestive association (1 time of false positive per GWAS), Genome-wise significance association (0.05 false positives per GWAS) and highly significance association (0.001 false positives per GWAS). The threshold P value of the levels of significance was calculated based on the estimated number of independent markers and LD blocks for the autosomal markers [20]. LD block was defined based on the ‘solid spine of LD’ algorithm with contiguous SNPs having pairwise D' values of greater than 0.8 calculated by PLINK. Using this approach, the estimated number of independent SNP markers and LD blocks was 97,358. Therefore, the significant threshold P values were 1.027E-05 (1/97,358) for suggestive or chromosome-wise significance, 5.130E-07 (0.05/97,358) for genome-wide significance and 1.027E-08 (0.001/97,358) for highly significant. Empirical genome wide P values were obtained from 1000,000 permutations for each SNP using the perm function in PLINK. Overview of SNP effects by Manhattan plots was produced by R studio (Version 0.98.994).

### Functional analysis

To analyze genes, their locations, and the position of SNP on the map, the UCSC Genome Browser on Chicken Nov. 2011 (ICGSC Gallus_gallus-4.0/galGal4) assembly implemented at https://www.genome.ucsc.edu/cgi-bin/hgGateway was used [21]. Information about genes provided from Refseq in NCBI (http://www.ncbi.nlm.nih.gov) based on Gene ID.

### Replication association studies

#### Chicken populations, samples and phenotype

For the first replication study, blood and egg samples of 146 individuals (N146) aged 43 wk were collected from the second generation derived from the former GWAS population of China Agricultural University Research Farm. Eggs were collected on 3 successive days to quantify the eggshell color traits QB, QP, and QT by the chemical method as was described above. All phenotype data were checked for normality before conducting any further analysis.

To test the marker effect on the eggshell blueness in a late stage of egg production, the second replication study was employed, in which blood and egg samples of 280 individuals (N280) aged 68 wk were collected from a separate population of Dongxiang chicken kept in Jiangxi Donghua Livestock & Poultry Breeding Co. Ltd. (Jiangxi Province, China). Eggs were collected on 3 successive days to quantify the eggshell color traits with the same measurement procedure previously described. All phenotype data were checked for normality before performing any further analysis.

The two repeat populations were fed the same type of the commercial diet, and the feeding protocol was provided by Zhengda Feed Co. Ltd. The main nutritional components are presented in S2 Table.

#### Genotyping

Genomic DNA was extracted from the blood samples of the two populations using TIANamp Blood DNA Kit (Tiangen, China), and a NanoDrop spectrophotometer (NanoDrop Technologies,USA) was used to measure its quantity and quality. Concentrations of DNA were adjusted to 50ng/μl. A set of SNPs distributed on GGA21 that anchored by GWAS in genome and chromosome-wise significance associated with QB, QP and QT were carefully examined. Twenty-four different SNP loci were realized for genotyping using matrix assisted laser desorption-ionization time-of-flight mass spectrometry (MALDI-TOF MS) on the Mass ARRAY iPLEX Platform (Sequenom, San Diego, CA).

#### Quantitative real-time PCR

In the N146 population, a total of 10 hens laying dark blue eggs and 10 hens laying light blue eggs were selected according to eggshell color. We recorded the oviposition time for each hen every one hour from 6:00 am to 6:00 pm every day. Five chickens laying dark blue eggs (DB) and five chickens laying light blue eggs (LB), with fairly uniform oviposition time, were slaughtered 3–5 h before the next expected oviposition; shell gland of the uterus was collected and frozen immediately in liquid nitrogen pending the expression analysis. In case of N280 population, also five chickens laying dark blue eggs (DB) and five chickens laying light blue eggs (LB), followed the same treatment as described above. Total RNA was extracted from the shell gland sample using TRIZOL Reagent (Invitrogen, San Diego, CA, USA), following the manufacturer’s protocol. RNA quantity and quality were detected by a NanoDrop spectrophotometer at 260/280 nm and the integrity of RNA was monitored on 1% agarose gel. The first strand of cDNA was synthesized from 2μg of purified total RNA using the Promega ImProm-IITM Reverse Transcription System (Beijing, China). The gene specific primers of five candidate genes Adherens junctions associated protein 1 (*AJAP1*), Tumor necrosis factor receptor superfamily, member 9 (*TNFRSF9*), Chromosome 1 open reading frame 174 (*C1ORF174*), Calmodulin binding transcription activator 1 (*CAMTA1*), and Centrosomal protein 104 (*CEP104*) were designed using Primer Premier 5.0 software (Premier Biosoft International, Palo Alto, CA, USA) based on the predicted *Gallus gallus* sequence of these genes deposited in the GenBank database. The real-time PCR efficiency of each pair of primers was calculated using 5 points in a 5-fold dilution series of cDNA, which was used to construct a standard curve. The house-keeping gene glyceraldehyde-3-phosphate dehydrogenase (*GAPDH*) was selected as internal standard. Primer sequences are listed in S3 Table. The qRT-PCR was conducted using two groups of dark and light blue eggshell with the following program: 95°C for 30 sec, 39 cycles at 95°C for 10 sec, 58°C for 30 sec, and a final extension step at 72°C for 10 sec on a CFX96 Real-time System (Bio-Rad, USA). Gene expression was normalized using the 2¯^ΔCT^ method. Data were analyzed by Student’s t-test to compare the differences of mRNA expression level between the two groups of DB & LB.

#### Statistical analysis for association study

The SNPs were first analyzed for allele and genotype frequency within each population by the chi-squared test. The SNPs that deviated from Hardy-Weinberg equilibrium were removed from the analysis. The SNP with a genotype call rate <85% and a minor allele frequency <1% over all individuals in both populations were also discarded from further analysis. Linkage disequilibrium (LD) between SNPs and haplotype blocks were assessed using the software Haploview4.2 (Broad Institute of MIT and Harvard, Cambridge, MA, USA). The association analysis between the identified SNPs or haplotype and phenotypic traits was carried out using the general linear model procedure in SAS version 9.1.3 (SAS Institute Inc. Cary, NC, USA) with the following fixed effect model: Y_ijk_ = μ+G_j_ +e_ijk_, where Y_ijk_ represents the phenotypes of QB, QP, and QT. μ is overall mean. G_j_ is the fixed effect of genotype or haplotype. e_ijk_ is the random residual error. Least-square means among different genotypes within each SNP or haplotype combination genotype were compared using the Duncan test. In the Model, the estimated genotype effect was further partitioned into additive effect (A) and dominant effect (D). The additive (a), dominant (d) effects of each SNP were calculated based on the equations proposed by Falconer et al. [22]: A = (AA˗BB)/2, D = AB ˗ (AA + BB)/2, where AA, AB and BB were least square means of genotypes AA, AB and BB, respectively.

## Results

### Phenotype and SNP data of GWAS

Phenotype data of four traits of quantities of biliverdin (QB), protoporphyrin (QP), quantity of the total pigments (QT) and color density (CD) were analyzed and presented in Table 1. Phenotypic data for all traits except CD were not normally distributed and Johnson transformation was used to achieve normality before conducting association analysis.

**Table 1:** Basic statistics of phenotype data for eggshell color.

Phenotype	N	Mean	SE	SD	Min	Max
QB^1^	139	4.97	0.17	1.96	1.04	11.63
QP^2^	139	2.03	0.10	1.14	0.44	7.83
QT^3^	139	7.00	0.24	2.87	2.30	18.50
CD	139	25.14	0.27	5.54	12.00	42.00

^1^ Quantity of biliverdin (×10^−8^ mol/g)

^2^ Quantity of protoporphyrin (×10^−8^ mol/g)

^3^ Total quantity of eggshell pigments (QT = QB + QP) (×10^−8^ mol/g)

4 Color density (CD) = 100-(color reflectance) (%).N (number of samples).

### Identification of SNP loci at genome or chromosome-wise significant level

In this study, genomic inflation factors of the population for four traits were estimated and the data are presented in S4 Table. Before inclusion PCA components as covariates in the association model, the inflation factor calculated for the genomic control analysis was more than 2 for all traits (that should be 1 under the null), indicating that population stratification is detected in our GWA data. Then, we calculated the inflation factor for the genomic control analysis for each trait after inclusion PCA components as covariates in the model to confirm the robustness of our analysis. The results showed values equal or close to 1.0 (S4 Table), which is consistent with the idea that there was some substructure inflating the distribution of test statistics in the previous analysis. Therefore, association tests were performed for the population using least squares regression analysis with PCA components as covariates for quantitative traits as suggested by Wang et al. [14]. Since Bonferroni correction is excessively conservative and can result in high proportion of negative false as marker density increase [23], we tested association at chromosome-wise significance level with P value thresholds ranging from 6.019E-05 on GGA1 to 9.091E-02 on LGE64 linkage group (S5 Table).

**Fig 1:**
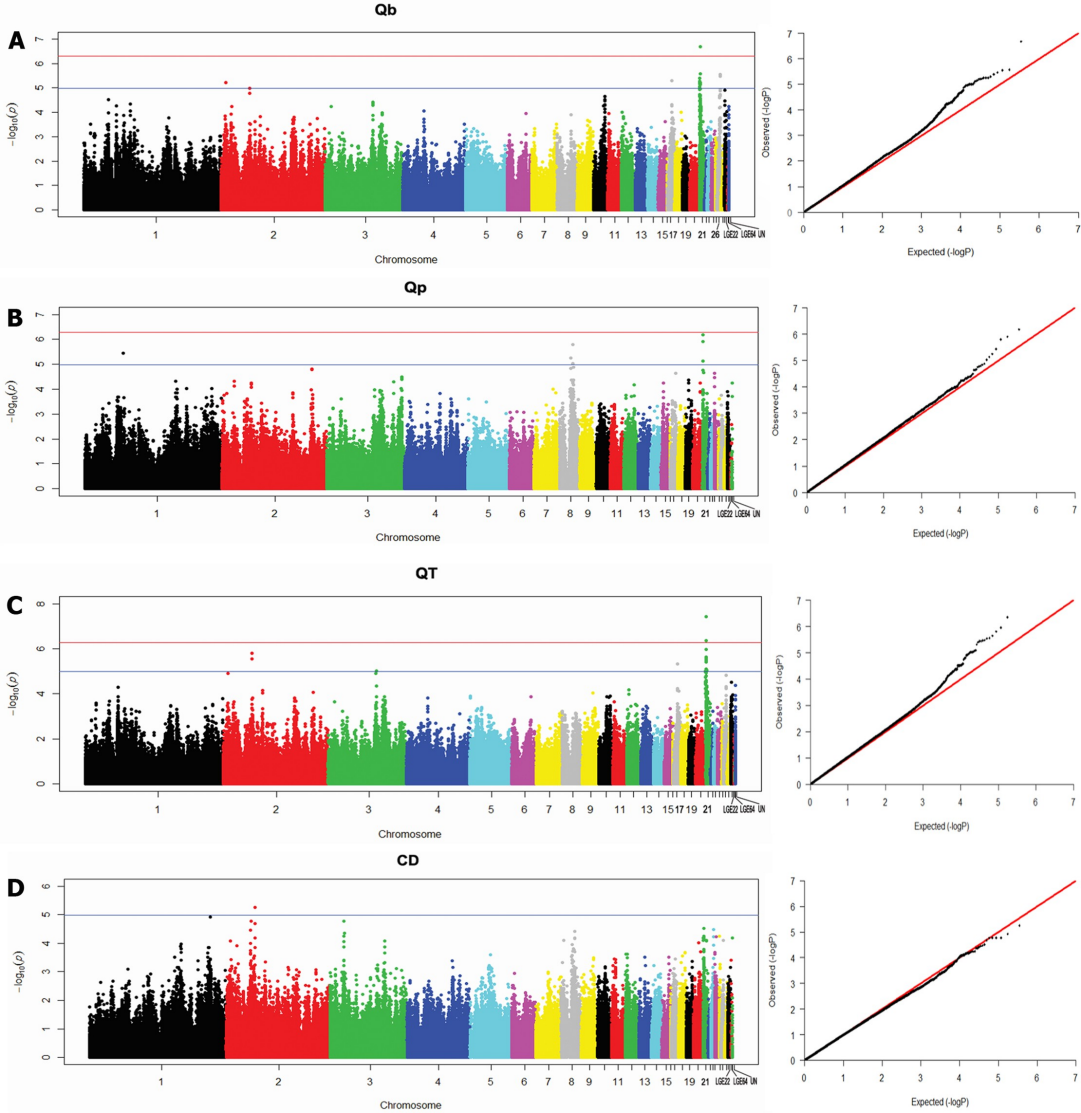
**Manhattan plot (left) and quantile-quantile plot (right) of the observed P-values for QB (A), QP (B), QT (C) and CD (D)**. In Manhattan plot, the red and gray horizontal lines indicate the genome-wise significance threshold [-log10 (5.135E-07)] and suggestive significance threshold [-log10 (1.027E-05)], respectively.

A total of 49 SNP effects (some of which are repeated within the traits) involving 35 different SNP markers and 29 genes were found to be associated with the four traits. Of the 49 SNP effects, three (2 SNP markers within or near 2 genes) on GGA21 reached 5% Bonferroni genome-wise significance in a P value threshold of 5.130E-07. All these significant SNPs reached 5% empirical genome-wise significance from 1,000,000 permutation tests. Moreover, forty-six SNP effects (35 SNP markers within or near 29 genes) reached the significance of suggestive linkage (P < 1.027E-05) distributing on seven chromosomes including GGA1, GGA2, GGA3, GGA8, GGA17, GGA21 and GGA26 under the LD condition. Of the 37 SNP markers, 24 SNP were located either within intron/exon or near 15 genes in a narrow ~1.17 Mb (135,295–1,306,399 bp) region of GGA21. The quantile-quantile plots and the Manhattan plot of P values, in terms of -log (P), for all SNP markers and different traits are presented in Fig 1.

Considering P value of 5.130E-07 as the genome-wise significance level with Bonferroni correction, it was revealed that two SNP markers on GGA21 showed significant association with QT and QB phenotypes of which, SNP rs315477097 significantly associated with QT and QB while SNP rs13602462 only related to QT. No SNPs were found to be associated with QP and CD traits at the genome-wise significance level (P < 5.130E-07). However, the number of SNP effects with suggestive significance (chromosome-wise significance) was much larger than those with genome-wise significance. The details of all genome-wise significant and suggestive significant SNPs, including their positions in the genome, P value, and candidate genes are summarized in Table 2.

**Table 2:** Associated SNPs with genome-wise significance and suggestive association (chromosome-wise significance) for eggshell color traits.

Row	Trait	SNP ID	GGA	Pos (bp)	^1^Nearest Gene	SNP	P value	^2^EMP1
1*	QT	rs315477097	21	819014	Intro 2 *AJAP1*	C/G	3.827E-08	1.00E-06
2*	QT	rs13602462	21	884536	60 Kb D *AJAP1*	C/T	4.400E-07	2.00E-06
					30 Kb U *C1ORF174*			
3*	QB	rs315477097	21	819014	Intro 2 *AJAP1*	C/G	2.093E-07	1.00E-06
4	QT	rs315586328	21	482280	6.6 Kb D *CAMTA1*	A/T	1.10E-06	1.00E-06
5	QT	rs313176000	2	41158174	30.2 Kb D *TRIM71*	A/G	1.55E-06	3.00E-06
					36Kb U *TCAIM*			
6	QT	rs315026428* *	21	482709	7.1 Kb D *CAMTA1*	C/T	2.28E-06	3.00E-06
7	QT	rs316634461	2	41153238	25.3 Kb D *TRIM71*	A/G	2.77E-06	3.00E-06
					36Kb U *TCAIM*			
8	QT	rs16177221	21	928230	Exon 12 *CEP104*	A/G	2.84E-06	6.00E-06
9	QT	rs313199923	21	838232	13.6 D *AJAP1*	C/T	3.29E-06	2.00E-06
10	QT	rs314071117https://www.ncbi.nlm.nih.gov/projects/SNP/snp_ref.cgi?rs=314071117	21	1087614	Intron 3 *PRDM16*	C/T	3.46E-06	4.00E-06
11	QT	rs16177219	21	925100	Exon 8 *CEP104*	G/T	3.55E-06	7.00E-06
12	QT	rs316040471	21	926781	Intron 9 *CEP104*	G/T	3.55E-06	7.00E-06
13	QT	rs315452432	21	995459	Intron 1 *TPRG1L*	A/G	3.93E-06	4.00E-06
					Intron 1 *MEGF6*			
14	QT	rs312758085	17	6324080	5.4Kb D *RAPGEF1*	C/T	4.83E-06	8.00E-06
					11.9Kb U *MED27*			
15	QT	rs16177211	21	917696	710bp D *C1ORF174*	A/G	8.08E-06	1.10E-05
16	QT	rs16177126	21	860224	35.6 D *AJAP1*	A/G	8.36E-06	1.40E-05
17	QT	rs15180893	21	1306399	44.2Kb D *ACTRT2*	A/G	9.13E-06	9.00E-06
18	QT	rs312869311	21	264776	Intron 5 *DJ-1*	C/T	9.49E-06	1.50E-05
19	QT	rs316706283	21	139487	Intron 12 *PADI1*	C/G	9.52E-06	1.50E-05
20	QT	rs317327045* *	3	68214333	Intron 13 *PREP*	G/T	9.60E-06	9.00E-06
21	QT	rs16177212	21	917720	734bp D *C1ORF174*	A/G	1.01E-05	1.10E-05
22	QB	rs13602462	21	884536	60 Kb D *AJAP1*	C/T	2.63E-06	2.00E-06
					30 Kb U *C1ORF174*			
23	QB	rs314959433	26	4009137	12.7 Kb U *FANCE*	A/G	2.81E-06	1.50E-05
					35.5 Kb D *NGF*			
24	QB	rs317648652	26	4142227	Intron 14 *ANKS1A*	A/G	3.37E-06	6.11E-05
25	QB	rs315026428* *	21	482709	7.1 Kb D *CAMTA1*	C/T	4.01E-06	3.00E-06
26	QB	rs312758085	17	6324080	5.4Kb D *RAPGEF1*	C/T	4.99E-06	8.00E-06
					11.9Kb U *MED27*			
27	QB	rs315586328	21	482280	6.6 Kb D *CAMTA1*	A/T	5.49E-06	1.00E-06
28	QB	rs316706283	21	139487	Intron 12 *PADI1*	C/G	5.54E-06	1.50E-05
29	QB	rs312869311	21	264776	Intron 5 *DJ-1*	C/T	5.59E-06	1.50E-05
30	QB	rs315327687	2	6569681	Exon 31 *KMT2C*	A/G	6.04E-06	1.00E-05
31	QB	rs313867043	21	271281	Intron 2 *TNFRSF9*	A/G	6.20E-06	1.70E-05
32	QB	rs16177221	21	928230	Exon 12 *CEP104*	A/G	6.26E-06	6.00E-06
33	QB	rs316919101	21	135295	13bp D *PADI3*	A/G	6.96E-06	2.20E-05
34	QB	rs16177219	21	925100	Exon 8 *CEP104*	G/T	7.42E-06	7.00E-06
35	QB	rs316040471	21	926781	Intron 9 *CEP104*	G/T	7.42E-06	7.00E-06
36	QB	rs15180009	21	147298	Intron 1 *PADI1*	C/T	8.66E-06	3.30E-05
37	QB	rs316597023	21	144447	Intron 3 *PADI1*	A/G	9.71E-06	3.00E-05
38	QB	rs317426373	21	159764	Intron 14 *PADI2*	C/T	9.71E-06	3.00E-05
39	QB	rs312427657	21	162882	Intron 1 *SDHB*	C/T	9.71E-06	3.00E-05
40	QB	rs316267959	21	170654	236bp D *SDHB*	C/T	9.71E-06	3.00E-05
					479 bp U *MRPS16*			
41	QB	rs317187121	21	264415	Intron 5 *DJ-1*	A/G	9.71E-06	3.00E-05
42	QP	rs13602462	21	884536	60 Kb D *AJAP1*	C/T	6.65E-07	1.00E-06
					30 Kb U *C1ORF174*			
43	QP	rs315477097	21	819014	Intro 2 *AJAP1*	C/G	1.24E-06	2.00E-06
44	QP	rs314697018	8	19253362	Intron 12 *IPO13*	C/T	1.59E-06	3.00E-06
45	QP	rs14827543* *	1	55010569	6.3 Kb D *STAB2*	C/T	3.59E-06	5.00E-06
46	QP	rs16633947	8	15657173	28.8 Kb U *TTLL7*	A/G	5.65E-06	5.00E-06
					36.6 Kb D *PRKACB*			
47	QP	rs313199923	21	838232	13.6 D *AJAP1*	C/T	7.32E-06	8.00E-06
					76.3 Kb U *C1ORF174*			
48	QP	rs16637835	8	19180600	Intron 2 *ST3GAL3*	A/G	9.43E-06	1.60E-05
49	CD	rs313176000	2	41158174	30.2 Kb D *TRIM71*	A/G	5.58E-06	5.00E-06
					36Kb U TCAIM			

* SNPs reached 5% Genome-wise significance level.

^1^D: downstream, U: upstream.

^2^Empirical P value by 1,000,000 permutation tests.

### Quantity of biliverdin (QB)

In genome-wise significance level, one SNP on GGA21 was found to be significantly associated with QB trait. SNP rs315477097 (P = 3.827E-08) was located in the Intron 2 of *AJAP1* gene. In chromosome-wise significance level (P < 1.027E-05), 20 SNPs were detected for QB phenotype, of which 16 SNPs were located within a ~793 Kb segment (between 135 Kb to 928 Kb) on GGA21, and four SNPs were distributed on three chromosomes including GGA2 (n = 1), GGA17 (n = 1), and GGA26 (n = 2). Of the 16 SNPs detected on GGA21 associated with QB phenotype, 11 were located in the intron or exon of five known genes (i.e. *PDAI1*, *SDHB*, *DJ-1*, *TNFRSF9* and *CEP104*), whilst five SNPs were positioned in the neighboring of six known genes (i.e. *PADI3*, *SDHB*, *MRPS16*, *CAMTA1*, *AJAP1* and *C1ORF174*) (Table 2).

### Quantity of protoporphyrin (QP)

For QP phenotype, the P values of association tests did not reach the 5% Bonferroni genome-wise significant level (P < 5.130E-07), but some SNP-trait combinations showed suggestive evidence with chromosome-wise significant level (P < 1.027E-05). In total, seven SNPs distributing on GGA1 (n = 1), GGA8 (n = 3) and GGA21 (n = 3) were detected to be associated with QP phenotype in chromosome-wise significance level (Table 2).

### Quantity of total eggshell pigments (QT)

In genome-wise significance level, two SNPs on GGA21 were found to be significantly associated with QT phenotype. Interestingly, the same SNP rs315477097 on GGA21 at position of ~819 Kb and in the Intron2 of *AJAP1* gene which was associated with QB trait in genome-wise significance level, also found to be significantly associated with QT phenotype (P = 2.09E-07), the other SNP rs13602462 was located at 60 Kb downstream region of *AJAP1* gene and 30 Kb upstream of *C1ORF174* gene (P = 4.400E-07) (Table 2).

### Color density (CD)

Similar to QP, the P values of the CD trait of association tests did not reach the 5% Bonferroni genome-wise significant level (P < 5.130E-07). However, the same SNP rs313176000 on GGA2 which showed suggestive significant association with QT phenotype, also was correlated with CD trait (P = 5.58E-06) (Table 2).

### Replication association analysis

#### Data filtering

In the current work, 24 SNP markers distributed on GGA21 showing significance in genome and chromosome-wise levels of GWAS were selected for genotyping using (MALDI-TOF MS). Initially, six SNPs were excluded for lack of genotypic data and low signal intensity. Thereafter, 18 SNP markers were successfully genotyped, of which 6 SNPs with genotype call rate <85% and minor allele frequency <1% over all individuals were discarded from the N146 and N280 populations (Table 3). In addition, the chi-square test showed that genotype frequencies of all SNPs in the two populations were in Hardy–Weinberg equilibrium except for one locus in N146 and 2 SNP loci in N280 which were also removed from any further analysis. In total, 11 and 10 SNP markers were subjected to the association analysis in both of the two populations, respectively. The information about SNP markers is shown in Table 4.

**Table 3:** Twenty-four SNP selected from GWAS for genotyping.

Row	SNP ID	SNP	GGA	Pos (bp)	Nearest Gene
1	rs315477097	C/G	21	819014	Intro 2 *AJAP1*
2	rs13602462	C/T	21	884536	60 Kb D *AJAP1* 30 Kb U *C1ORF174*
3	rs315026428**** ***	C/T	21	482709	7.1 Kb D *CAMTA1*
4	rs315586328	A/T	21	482280	6.6 Kb D *CAMTA1*
5	rs316706283	C/G	21	139487	Intron 12 *PADI1*
6	rs312869311	C/T	21	264776	Intron 5 *DJ-1*
7	rs313867043	A/G	21	271281	Intron 2 *TNFRSF9*
8	rs16177221	A/G	21	928230	Exon 12 *CEP104*
9	rs316919101	A/G	21	135295	13bp D *PADI3*
10	rs16177219	G/T	21	925100	Exon 8 *CEP104*
11	rs316040471	G/T	21	926781	Intron 9 *CEP104*
12	rs15180009* *	C/T	21	147298	Intron 1 *PADI1*
13	rs316597023*	A/G	21	144447	Intron 3 *PADI1*
14	rs317426373	C/T	21	159764	Intron 14 *PADI2*
15	rs312427657*	C/T	21	162882	Intron 1 *SDHB*
16	rs316267959	C/T	21	170654	236bp D *SDHB* 479 bp U *MRPS16*
17	rs317187121	A/G	21	264415	Intron 5 *DJ-1*
18	rs313199923	C/T	21	838232	13.6 D *AJAP1*
19	rs16177126* *	A/G	21	860224	35.6 D *AJAP1*
20	rs16177211*	A/G	21	917696	710bp D *C1ORF174*
21	rs16177212	A/G	21	917720	734bp D *C1ORF174*
22	rs315452432*	A/G	21	995459	Intron 1 *TPRG1L*, intron 1 *MEGF6*
23	rs314071117https://www.ncbi.nlm.nih.gov/projects/SNP/snp_ref.cgi?rs=314071117	C/T	21	1087614	Intron 3 *PRDM16*
24	rs15180893*	A/G	21	1306399	44.2Kb D *ACTRT2*

* These SNPs were initially excluded from genotyping process; the remaining SNPs were successfully genotyped

**Table 4:** SNP markers involved in the association analysis for N146 and N280 populations.

Row	SNP ID	SNP	GGA	Pos (bp)	Nearest Gene
1	rs315477097	C/G	21	819014	Intro 2 *AJAP1*
2^#^	rs13602462	C/T	21	884536	60 Kb D *AJAP1* 30 Kb U *C1ORF174*
3	rs315586328	A/T	21	482280	6.6 Kb D *CAMTA1*
4	rs316706283	C/G	21	139487	Intron 12 *PADI1*
5	rs313867043	A/G	21	271281	Intron 2 *TNFRSF9*
6*	rs316919101	A/G	21	135295	13bp D *PADI3*
7	rs16177219	G/T	21	925100	Exon 8 *CEP104*
8	rs15180009* *	C/T	21	147298	Intron 1 *PADI1*
9	rs313199923	C/T	21	838232	13.6 D *AJAP1*
10	rs16177126* *	A/G	21	860224	35.6 D *AJAP1*
11	rs16177212	A/G	21	917720	734bp D *C1ORF174*
12*	rs314071117https://www.ncbi.nlm.nih.gov/projects/SNP/snp_ref.cgi?rs=314071117	C/T	21	1087614	Intron 3 *PRDM16*

^#^ This SNP is involved in the association analysis of the N280 population but deleted in N146 population for the deviation of Hardy–Weinberg equilibrium

* This SNP is involved in the association analysis of N146 population but deleted in N280 for the deviation of Hardy–Weinberg equilibrium

#### Association analysis and SNP effect

Phenotype data for the 3 traits included in this study were normally distributed. Descriptive statistics of phenotypic measurements QB, QP, and QT in N146 and N280 populations are given in Table 5.

**Table 5:** Descriptive statistics of phenotypic data for eggshell color intensity traits.

Population	Phenotype	N	Mean	SE	SD	Min	Max
N146	QB^1^	146	34.37	0.86	10.40	13.63	69.47
	QP^2^	146	3.86	0.07	0.91	2.17	8.17
	QT^3^	146	38.23	0.90	10.86	15.80	73.51
N280	QB^1^	280	11.08	0.15	2.53	5.84	21.41
	QP^2^	280	4.06	0.08	1.32	1.84	8.75
	QT^3^	280	15.14	0.21	3.53	8.15	28.69

^1^ Quantity of biliverdin (×10^−8^ mol/g)

^2^ Quantity of protoporphyrin (×10^−8^ mol/g)

^3^ Total quantity of eggshell pigments (QT = QB + QP) (×10^−8^ mol/g). N (number of samples).

In the N146 population, statistical analysis revealed that out of eleven SNPs, four SNPs (rs313867043, rs16177219, rs16177126, and rs16177212) were significantly associated with QB and QT traits (P < 0.05), but none of the SNPs were related to QP (P > 0.05). Chickens with the homozygous AA genotype were associated with higher QB and QT at SNP rs313867043 and SNP rs16177126 than were those with the homozygous GG or heterozygous GA genotype. At locus rs16177219 the homozygous GG was associated with higher QB and QT than were the genotypes GT and TT (P < 0.05), whilst, the homozygous GG at SNP rs16177212 was associated with higher QB and QT than were GA and AA genotype (P < 0.05) (Table 6).

**Table 6:** Effect of 11 SNPs on eggshell color intensity trait in N146 population.

SNP ID	Genotype	QB	QP	QT
rs315477097	GG (77)	34.71±1.19	3.86±0.10	38.57±1.24
	GC (61)	33.46±1.33	3.85±0.12	37.31±1.39
	CC (8)	38.06±3.68	3.94±0.32	42.01±3.85
	P value	0.4628	0.9641	0.4810
rs315586328	AA (118)	34.76±0.96	3.81±0.08	38.56±1.00
	AT (27)	32.63±2.01	4.10±0.18	36.74±2.10
	TT (1)	36.09±10.4	3.41±0.91	39.50±10.1
	P value	0.6272	0.2733	0.7310
rs316706283	CC (77)	34.83±1.18	3.87±0.10	38.70±1.23
	CG (60)	33.05±1.34	3.82±0.11	36.86±1.40
	GG (8)	40.30±3.67	4.09±0.32	44.38±3.83
	P value	0.1577	0.7251	0.1619
rs313867043	GG (82)	35.02±1.14^**ab**^	3.88±0.10	38.90±1.19^**ab**^
	GA (54)	32.54±1.41^**a**^	3.74±0.12	36.28±1.47^**a**^
	AA (6)	42.51±2.23^**b**^	4.25±0.37	46.76±2.41^**b**^
	P value	**0.0407**	0.3439	**0.0383**
rs316919101	AA (37)	34.55±1.70	3.74±0.15	38.29±1.78
	AG (78)	33.11±1.17	3.91±0.10	37.03±1.22
	GG (31)	37.33±1.86	3.86±0.16	41.19±1.94
	P value	0.1605	0.6456	0.1969
rs16177219	GG (44)	36.73±1.55^**a**^	3.93±0.14	40.66±1.62^**a**^
	GT (78)	32.45±1.16^**b**^	3.84±0.10	36.29±1.21^**b**^
	TT (24)	36.28±2.09^**ab**^	3.79±0.19	40.07±2.19^**ab**^
	P value	**0.0361**	0.7754	**0.0468**
rs15180009	CC (37)	34.55±1.70	3.74±0.15	38.29±1.78
	CT (78)	33.11±1.17	3.91±0.10	37.03±1.22
	TT (31)	37.33±1.86	3.86±0.16	41.19±1.94
	P value	0.1605	0.6456	0.1969
rs313199923	CC (50)	34.08±1.48	3.79±0.13	37.88±1.55
	CT (75)	34.80±1.21	3.86±0.10	38.67±1.2
	TT (20)	33.61±2.35	4.03±0.20	37.64±2.45
	P value	0.8744	0.6289	0.8920
rs16177126	GG (70)	35.62±1.32^**ab**^	4.18±0.18	39.79±1.53^**ab**^
	GA(67)	31.19±1.94^**a**^	3.94±0.17	34.47±1.20^**a**^
	AA (9)	39.77±1.27^**b**^	3.28±0.23	43.71±1.48^**b**^
	P value	**0.0388**	0.1049	**0.0478**
rs16177212	GG (43)	37.04±1.56^**a**^	3.95±0.13	40.99±1.63^**a**^
	GA (79)	32.34±1.15^**b**^	3.83±0.10	36.17±1.20^**b**^
	AA (24)	36.28±2.09^**ab**^	3.79±0.18	40.07±2.18^**ab**^
	P value	**0.0345**	0.7328	**0.0413**
rs314071117	CC (67)	34.49±1.27	3.90±0.11	38.39±1.33
	CT (65)	34.84±1.29	3.88±0.11	38.72±1.35
	TT (14)	31.65±2.79	3.52±0.24	35.17±2.91
	P value	0.5814	0.3424	0.5362

QB, QP, and QT (×10^−8^ mol/g).

^**a**^, ^**b**^ within the same column with different superscripts means P < 0.05. Values in bold indicate significant association with the trait

For N280 population, statistical analysis showed that out of ten SNPs, two SNPs (rs16177219 and rs16177126) were significantly associated with QB and QT traits. Polymorphism rs315477097 was strongly associated with QB, QP and QT traits, while Polymorphism rs13602462 was significantly correlated with the 3 phenotype traits. SNP rs315586328 and rs313199923 were strongly associated with QB (P < 0.01) and significantly with QT, while SNP rs16177212 significantly related only to QP (P < 0.05) (Table 7). At locus rs315477097, individuals with the homozygous GG associated with higher QB, QP, and QT. The homozygous genotype CC at locus rs13602462 was associated with higher QB and QT, while genotype CT associated with higher QP. At locus rs315586328 genotype AA associated with higher QB and QT whereas genotype TT in polymorphism rs313199923 associated with higher levels of both traits. Interestingly, two mutual SNP loci (rs16177219 and rs16177126) were discovered between this population and N146 population in which the same homozygous genotype GG and AA were significantly associated with increased levels of QB and QT in both of the two SNPs, respectively. Genotype GG in the polymorphism rs16177212 was significantly attached to higher QP.

**Table 7:** Effect of 10 SNPs on eggshell color intensity trait in N280 population.

SNP ID	Genotype	QB	QP	QT
rs315477097	GG (130)	13.51±1.84^**A**^	4.41±0.92^**a**^	17.93±2.59^**A**^
	GC (113)	8.02±1.21^**B**^	2.41±0.60^**b**^	10.42±1.70^**B**^
	CC (37)	8.40±1.84^**AB**^	3.12±0.92^**ab**^	11.51±2.60^**AB**^
	P value	**0.0008**	**0.0045**	**0.0007**
rs13602462	CC (121)	11.55±1.31^**ab**^	3.64±0.65^**ab**^	15.39±1.85^**ab**^
	CT (123)	11.32±1.17^**a**^	3.96±0.58^**a**^	15.28±1.65^**a**^
	TT (35)	7.05±1.63^**b**^	2.34±0.81^**b**^	9.39±2.30^**b**^
	P value	**0.0257**	**0.0444**	**0.0228**
rs315586328	AA (211)	11.66±0.88^**A**^	3.30±0.44	14.97±1.24^**a**^
	AT (65)	10.09±0.88^**B**^	3.32±0.44	13.42±1.25^**b**^
	TT (4)	8.17±2.07^**AB**^	3.31±1.04	11.48±2.92^**ab**^
	P value	**0.0024**	0.9954	**0.0494**
rs316706283	CC (136)	10.11±1.45	3.69±0.72	13.80±2.03
	CG (114)	10.33±1.23	3.33±0.61	13.67±1.73
	GG (30)	9.48±1.71	2.92±0.86	12.40±2.42
	P value	0.8715	0.7109	0.8934
rs313867043	GG (92)	10.01±1.26	3.31±0.63	13.32±1.78
	GA (138)	10.42±1.25	3.54±0.63	13.96±1.77
	AA (32)	9.49±1.63	3.09±0.82	12.57±2.30
	P value	0.7028	0.6583	0.6550
rs16177219	GG (184)	14.01±1.81^**a**^	4.94±0.87	18.90±2.53^**a**^
	GT (86)	10.63±1.18^**ab**^	3.59±0.56	14.24±1.64^**ab**^
	TT (10)	6.60±1.89^**b**^	2.82±0.90	9.42±2.64^**b**^
	P value	**0.0274**	0.0637	**0.0244**
rs15180009	CC (125)	9.95±1.08	3.35±0.535	13.30±1.51
	CT (117)	10.47±1.13	3.48±0.564	13.95±1.59
	TT (38)	9.50±1.18	3.11±0.590	12.61±1.66
	P value	0.1152	0.3256	0.1345
rs313199923	CC (86)	4.84±2.16^**a**^	1.63±1.08	6.48±3.05^**a**^
	CT (146)	9.38±1.13^**b**^^**A**^	3.24±0.57	12.62±1.60^**b**^
	TT (48)	15.70±2.17^**b**^^**B**^	5.07±1.09	20.76±3.06^**c**^
	P value	**0.0099**	0.1941	**0.0216**
rs16177126	GG (239)	4.29±2.04^**a**^	2.31±1.02	6.61±2.88^**a**^
	GA (39)	9.34±0.91^**b**^	3.40±0.46	12.74±1.29^**b**^
	AA (2)	16.29±3.32^**b**^	4.23±1.66	20.52±4.68^**b**^
	P value	**0.0206**	0.5126	**0.0405**
rs16177212	GG (177)	6.37±2.12	8.86±1.06^**a**^	7.26±2.99
	AG (91)	9.38±1.29	3.13±0.64^**b**^	12.51±1.82
	AA (12)	14.17±2.34	5.92±1.17^**ab**^	20.10±3.29
	P value	0.1332	**0.0320**	0.0645

QB, QP, and QT (×10^−8^ mol/g).

^**a**^, ^**b**^, ^**c**^ within the same column with different superscripts means P < 0.05.

^**A**^, ^**B**^ Within the same column with different superscripts means P < 0.01. Values in bold indicate significant association with the trait

#### Additive and dominant effect of SNPs

The genotypic effects of the SNPs in the two populations were further divided into additive and dominant effects. In the N146 population, SNP rs313867043, rs16177219 and rs16177212 showed significant dominant effects on QB and QT (P < 0.05) (S6 Table).

For N280, the additive effect of the SNP rs315477097 was significant for QB and QT, whereas its dominant effect was significant for QP (P < 0.05). The additive genotype effect of SNP rs315586328, rs15180009, and rs313199923 was significant for QB (P < 0.05), whereas the dominant effect of SNP rs16177212 was significant for QP (P < 0.05) (S7 Table).

#### Haplotype analysis

**Fig 2:**
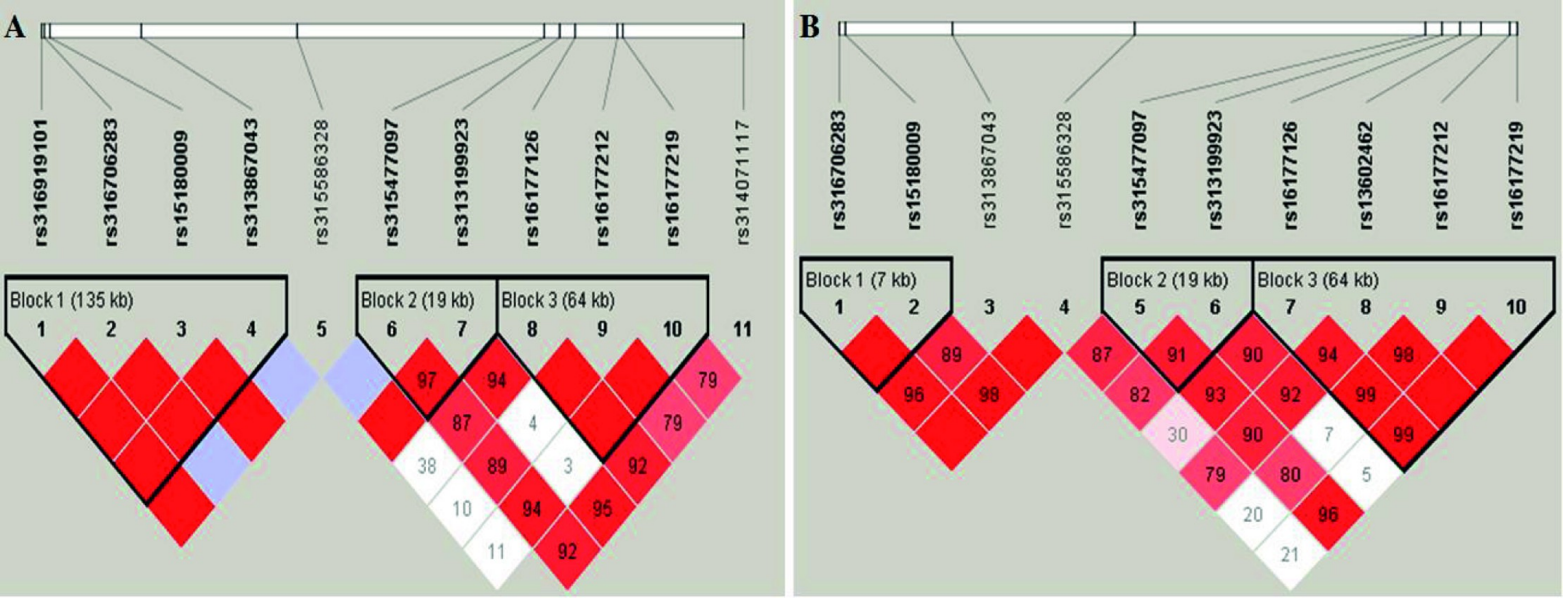
The haplotype blocks and linkage disequilibrium. (A) the N146 population, (B) the N280 population. The darker shading indicates higher linkage disequilibrium and the number within rhombus was D' value.

For the N146 population, three haplotype blocks were inferred as shown in Fig 2A by Haploview 4.2 in [24]. Block 1 consisted of 4 SNPs, including rs316919101, rs316706283, rs15180009, and rs313867043 with D’ of 1.00. Two SNPs rs315477097 and rs313199923 formed block 2 with D’ of 0.97. Block 3 was composed of 3 SNPs rs16177126, rs16177212, and rs16177219 and the value of D’ was 100. In the N280 population, also three haplotype blocks were detected as shown in Fig 2B. The first block consisted of 2 SNPs, rs316706283 and rs15180009 with D’ value equals to 1.00. Two SNPs rs315477097 and rs313199923, as well as 4 SNP rs16177126, rs13602462, rs16177212, and rs16177219 with D’ ranged from 0.91 to 0.99 were formed the second and third block, respectively.

Associations of the haplotypes with 3 eggshell color intensity traits in two populations are presented in the S8 and S9 Tables. In the N146 population, the data analysis revealed that the haplotype combinations in Block 1, 2 and 3 were not significantly associated with QB, QP and QT. However, multiple comparisons between the different combination genotypes were significant in block 1 for QB and QT. The greatest QB and QT values were observed with the genotype GCTGGGTG in block 1. For the N280 population, haplotype combinations in Block 2 and 3 were considerably associated with QP (P < 0.05) in which the combination CCCT in block 2 and the combination GCAGGTGG in block 3 were associated with the greatest QP. Multiple comparisons between the different combination genotypes were noticed significantly in block 1 for QB, and in block 2 and 3 for QB and QT. The combination genotype GTGT in block1 associated with greater QB, whereas the combinations CCCT in block 2 and GCAGGTGG in block 3 were higher in QB and QT.

#### Quantitative analysis of the candidate genes

**Fig 3:**
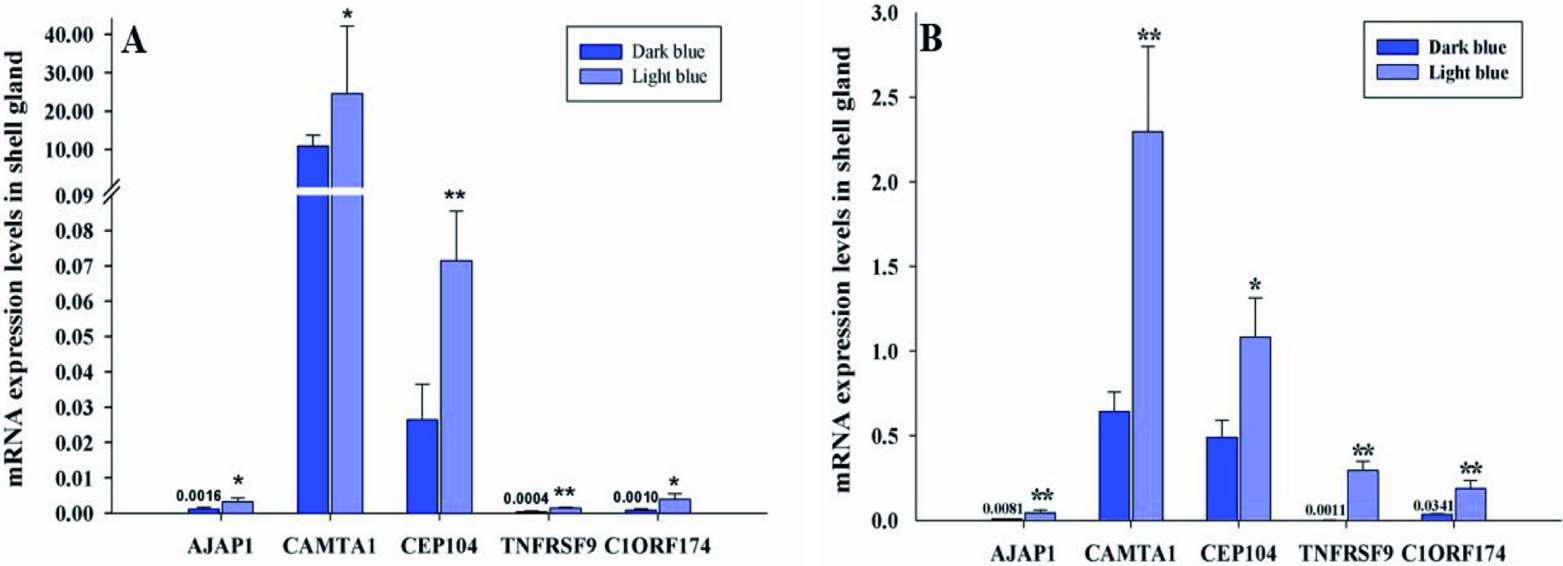
Expression levels of the five candidate genes *AJAP1*, *CAMTA1*, *CEP104*, *TNFRSF9*, and *C1ORF174* in dark and light blue-shelled chickens. (A) the N146 population, (B) the N280 population. Note error bars represent the mean ± SE. * denote the difference of expression level with significant difference (P < 0.05). ** denote the difference of expression level with significant difference (P < 0.01).

PCR efficiencies of the specific genes and *GAPDH* were within 95 to 105%, which was accepted for qRT-PCR. In this experiment, 5 candidate genes associated with the significant SNP loci were selected for detecting the mRNA expression levels between two groups of dark and light blue eggshell chickens. mRNA expression levels in all of the 5 candidate genes were shown down-regulated in dark blue than in light blue chickens in both of the N146 and N280 chicken populations and the differential expression was significantly different (Fig 3).

## Discussion

Genome wide association studies (GWASs) have been proven to be a powerful approach and efficient tool for genetic dissection of trait loci [25]. In chickens, the genomic regions influencing egg production and quality traits of White Leghorn and brown-egg dwarf layers [26] have been identified by GWASs. Eggshell color results from an accumulation of pigments on the eggshell. Among the eggshell pigments, biliverdin is responsible for the blue-green eggshell color, whereas protoporphyrin is associated with the reddish-brown color [27]. However, protoporphyrin and biliverdin co-exist in both blue eggshell and brown eggshell in different proportion. In the current study, we measured the quantity of biliverdin (QB) and protoporphyrin (QP) pigments, and total quantity of biliverdin and protoporphyrin pigments (QT) along with color density of eggshell surface (CD) in blue eggshell as phenotypic traits. In GWAS, due to the number of tests, the more relaxed the threshold in the discovery sample the more false positives will be discovered, the more stringent the significance threshold the more likely it is that real genetic effects of small size will be overlooked due to sampling effects [28]. Duggal et al. [29] examined the effective number of independent SNPs for GWAS panels and recommended using 10^−5^, 10^−7^ and 10^−8^ as "suggestive", "significant" and "highly significant" P value thresholds for the Affymetrix 500 K and Illumina 317 K GWAS SNP panels to properly control the family wide Type 1 error. They believe the suggestive association threshold should be used to identify SNPs for consideration in follow-up studies, and both the significant and highly significant associations should be considered regions more likely of association. Herein, a total of 49 SNP effects involving 35 different SNP markers and 29 genes were found to be associated with the four traits. From the 49 effects, 24 markers were observed in ~1.17 Mb (135,295–1,306,399 bp) region of GGA21. As quantity of biliverdin is an important criterion to determine the blue eggshell color intensity in blue-shelled chickens, 21 significant SNPs were detected to be associated with quantity of biliverdin. Of the 21 SNPs, one was in the genome-wise significance level and 20 SNPs showed suggestive significance, of which 16 SNPs were distributed on GGA21. The most significant SNP associated with quantity of biliverdin was found to be located in the intron 2 of *AJAP1* gene, also known as *MOT8*, *SHREW-1* and *SHREW1*, and this SNP is also observed in genome-wise significance level for QT trait.

To further narrow down the genomic region identified by our GWAS and verify the impact of the SNP marker on the eggshell color intensity traits, we have conducted a follow-up replication analysis in two different populations at two different age stages of Dongxiang chicken. In the first experiment, 4 SNP effects were found to be associated significantly with QB and QT of the eggshell at the age of 43 wk, but none of the SNPs were correlated with QP trait. The first SNP locus was located in Intron 2 of *TNFRSF9* also known as (*ILA*, *41BB*, *CD137* and *CDw137*), that is a protein-coding gene and the protein encoded by this gene is a member of the TNF-receptor superfamily. This receptor contributes to the clonal expansion, survival, and development of T cells. This gene plays a general potential role in immune function, and the expression of its receptor is induced by lymphocyte activation [30, 31]. The second SNP was located in 35.6 D *AJAP1*, a gene that is a novel transmembrane protein of adherent junctions in epithelial cells. *AJAP1* may affect cell motility, migration, invasion and proliferation by unclear mechanisms [32]. In polarized epithelial cells, *AJAP1* localizes and interacts with β-catenin in the E-cadherin-catenin complex and is found in cell-cell contacts in the human mammary gland, the uterus, and breast carcinoma cells [33]. The epigenetic silencing and deletion of *AJAP1* on human chromosome 1p36 was correlated with glioblastoma tumors and this loss of expression is due to promoter methylation [34]. The third SNP was located 734bp D *C1ORF174* gene. This gene is also found to be located on the deleted region of human chromosome 1p36 and associate with glioblastoma tumors and cell lines [35]. The fourth SNP found in this analysis was located in exon 8 of *CEP104* gene, this SNP was synonymous and caused no change in the amino acids of this gene. This gene is a protein coding gene that in humans encodes centrosomal protein 104 kDa protein. Like its Chlamydomonas ortholog, *FAP256*, it has been shown to localize to the distal ends of both centrioles in the absence of a cilium. During cilium formation, it is found at the tip of the elongating cilium [36]. For the N280 population, 6 SNP loci were associated significantly with QB, QP and QT of the eggshell at the age of 68 wk, in which two SNPs have shared their significant correlation in both of the two populations. One SNP locus was observed significantly associated with QB and QT. This SNP located 6.6 Kb D of *CAMTA1* gene. This gene localizes to 1p36 deletion region, it is a tumor suppressor candidate that inhibits key features of malignant cells and is involved in neuronal differentiation. Furthermore, The low expression of this gene is associated with neuroblastoma [37]. Taken together, the integrated genomic region that was significantly identified in the two different association analyses was narrowed down to ~ 653.819 Kb on Gallus gallus chromosome 21 and harbors 5 candidate genes. These genes were isolated initially from a region on chromosome 1p36 in human that is frequently deleted in neuroblastoma, glioblastoma and other tumor diseases [35]. Most of these genes have been studied in human but their functions are not fully identified in chickens. The association revealed in this study suggests the need for further investigation on the role of these five candidate genes in chicken eggshell blueness.

To shed light on the potential regulatory role that these candidate genes might play in blue eggshell color intensity traits, we further performed quantitative expression analysis in the shell gland of samples from two different populations. In this experiment, the 5 genes were shown significantly down-regulated in the dark blue eggshell chicken than that of the light blue group. In a recent research, Hötte et al. [38] documented that *AJAP1* is a putative negative regulator of angiogenesis in which the down regulation of *AJAP1* leads to an increase in the cumulative sprout length during sprouting angiogenesis; they found that the number of sprouts was increased upon *AJAP1* down regulation. In our qPCR analysis, *AJAP1* gene was shown down-regulated in the dark blue-shelled chicken in the two populations. In this regard, we hypothesize that the low expression level of *AJAP1* in endogenous cells of the shell gland in the dark group chicken has allowed more erythrocytes to flow through the wide blood vessels, and that led to a rise in the rate of heme synthesis resulted in an increased level in the production of biliverdin. It has been suggested that damaged and aging erythrocytes could be engulfed by macrophages in the shell gland [4, 8], and heme could be then oxidized to biliverdin or converted to protoporphyrin by substituting Fe2+ by H+. Zhao et al. [7] stated that biliverdin supposed to be synthesized in the shell gland and then deposited onto the eggshell of chickens. Here, we speculate that the higher expression levels of *TNFRSF9* gene in the light group chicken induced the activation of macrophages in the cells of shell gland in which a little heme was released from the disintegrated erythrocytes and this little amount of heme was further catabolised to the biliverdin or protoporphyrin that stimulated and simultaneously deposited on the eggshell in different ratios giving a pale pigment of the shell [3]. Furthermore, we have searched databases and related literature to find a detailed functional description for the differential mRNA expression of *CAMTA1*, *C1ORF174*, and *CEP104* genes between the dark and light blue eggshell chicken, but little evidence is available regarding their role in biliverdin or protoporphyrin pigment pathway.

Numerous studies attributed the cause of the eggshell color intensity to age, oxidative stress, diet contents, fear, and disease however; the potential regulatory genes and its related proteins and detailed molecular mechanisms regulating eggshell blueness have yet to be clearly defined [39, 40]. Although important genetic effects of pigment traits exist, the genetic architecture of the traits and its quantity is still poorly understood. Thus, identifying causal genes underlying these traits provide novel goals for estimation the hue of blue eggshell. With the development of high throughput SNP genotyping technologies, the GWAS has generally become a vital approach for investigating mutations underlying complex traits. Results from the GWAS have been efficiently applied in identifying functional genes involved in substantial economical traits in chickens [41].

In light of the data presented here, as a significant signal was found for the *AJAP1* gene in our GWAS, the *AJAP1* gene might be a target gene for biliverdin quantity traits in blue eggshell chickens. Our follow-up association analysis results also showed that *AJAP1* as well as *CEP104*, *C1ORF174*, *TNFRSF9*, and *CAMTA1* genes might be used as genetic markers with significant effects on selection strategy to obtain chickens that lay eggs with uniform colors which would benefit the breeders to conserve the uniform color for future breeding programs.

Specific function of these genes have not yet been determined clearly in chicken and to the best of our knowledge, there are no studies of the expression and regulatory mechanisms of *AJAP1*, *C1ORF174*, *CAMTA1*, *CEP104*, and *TNFRSF9* in chickens, including blue-shelled chickens. To address these issues, we examined their mRNA expression profile in the shell gland in order to identify their potential regulatory mechanism, however further studies are warranted to explore the regulation sites that might be altered by DNA methylation status or loss of heterozygosity, epigenetic modifications and deletions in each of dark and light blue-shelled chicken.

## Conclusion

Our GWA study revealed two genome-wise significant SNPs and 35 chromosome-wise significant SNPs besides 29 genes associated with eggshell color intensity traits, of which 24 SNPs and 15 genes were distributed in a relatively narrow region (~1.17Mb) of chicken chromosome 21. In the follow-up replication analysis, we verified the association of eight markers in a narrow region of 653.819 Kb with eggshell color intensity traits. This genomic region contained the candidate genes *AJAP1*, *C1ORF174*, *CAMTA1*, *CEP104*, and *TNFRSF9*. Further expression profile analysis indicated that these five genes significantly associated with dark and light blue egg shelled chickens. The associations of these genes with blue eggshell color intensity traits are reported here for the first time, further in-depth studies including using of protein and cell experiments are required for better understand the biological mechanism underlying chicken eggshell blueness.

## Supporting information

S1 Table Distributions of Affymetrix 600K Axion Chicken SNP array and their conditions after quality control. 1 Linkage group, 2 these SNPs are not assigned to any chromosomes.
(DOCX)

S2 Table Nutrition ingredients of the commercial diet for laying chickens. The values are given in (%). Met: methionine.
(DOCX)

S3 Table Primer pairs of the candidate genes used for quantitative real-time PCR. 1 GenBank Accession No. relates to the transcript used for the design of the primers.
(DOCX)

S4 Table Genomic inflation factors of the traits before and after inclusion PCA and MDS components as covariates in the linear squires regression model for GWAS. (DOCX)

S5 Table Chromosome-wise significant threshold for each chromosome. ^1^Linkage group. ^2^These SNPs are not assigned to any chromosomes.
(DOCX)

S6 Table Additive and dominant effect of SNPs in N146 population on QB, QP, and QT traits. QB, QP, and QT (×10^−8^ mol/g). Values in bold indicate significant association with the trait.
(DOCX)

S7 Table Additive and dominant effect of SNPs in N280 population on QB, QP, and QT traits. QB, QP, and QT (×10^−8^ mol/g). Values in bold indicate significant association with the trait.
(DOCX)

S8 Table Associations of the haplotype combinations with eggshell color intensity traits in the N146 population of Dongxiang chicken (LSM±SE). QB, QP, and QT (×10^−8^ mol/g). Means in the same column with different lowercase superscripts are different at P < 0.05.
(DOCX)

S9 Table Associations of the haplotype combinations with eggshell color intensity traits in the N280 population of Dongxiang chicken (LSM±SE). QB, QP, and QT (×10^−8^ mol/g). Means in the same column with different lowercase superscripts are different at P < 0.05. Values in bold indicate significant association with the trait.
(DOCX)
