# Supplementary material for: Genome-wide association study and a post replication analysis revealed a promising genomic region and candidate genes for chicken eggshell blueness
Source: PLoS One. 2019 Jan 23;14(1):e0209181. doi: 10.1371/journal.pone.0209181 (PMC6343938; doi:10.1371/journal.pone.0209181)
Supplement: S1 Table — 1 Linkage group, 2 these SNPs are not assigned to any chromosomes. (DOCX) [file pone.0209181.s001.docx]

**S1 Table.** Distributions of Affymetrix 600K Axion Chicken SNP array and their conditions after quality control

|  | Before quality control | | | After quality control | | |
| --- | --- | --- | --- | --- | --- | --- |
| Chromosome | Physical Map (Mb) | No. of SNP Markers | Marker Density (Kb/SNP) | Physical Map (Mb) | No. of SNP Markers | Marker Density (Kb/SNP) |
| 1 | 195.3 | 102351 | 1.9 | 195.2 | 62839 | 3.1 |
| 2 | 148.8 | 64435 | 2.3 | 148.8 | 41969 | 3.5 |
| 3 | 110.4 | 57233 | 1.9 | 110.4 | 36906 | 3.0 |
| 4 | 90.2 | 43337 | 2.1 | 90.2 | 27587 | 3.3 |
| 5 | 59.5 | 30616 | 1.9 | 59.5 | 20024 | 3.0 |
| 6 | 34.9 | 21943 | 1.6 | 34.9 | 14660 | 2.4 |
| 7 | 36.2 | 21604 | 1.7 | 36.2 | 14271 | 2.5 |
| 8 | 28.8 | 17274 | 1.7 | 28.7 | 10887 | 2.6 |
| 9 | 23.4 | 18117 | 1.3 | 23.4 | 11906 | 2.0 |
| 10 | 19.9 | 18947 | 1.1 | 19.9 | 11771 | 1.7 |
| 11 | 19.4 | 13984 | 1.4 | 19.4 | 8232 | 2.4 |
| 12 | 19.9 | 14829 | 1.3 | 19.8 | 8769 | 2.3 |
| 13 | 17.8 | 11282 | 1.6 | 17.8 | 7125 | 2.5 |
| 14 | 15.1 | 13181 | 1.1 | 15.1 | 8657 | 1.7 |
| 15 | 12.6 | 10505 | 1.2 | 12.6 | 6520 | 1.9 |
| 16 | 0.5 | 584 | 0.9 | 0.5 | 228 | 2.2 |
| 17 | 10.4 | 9379 | 1.1 | 10.3 | 5674 | 1.8 |
| 18 | 11.2 | 9673 | 1.2 | 11.2 | 6144 | 1.8 |
| 19 | 10 | 9044 | 1.1 | 10 | 5567 | 1.8 |
| 20 | 14.3 | 9614 | 1.5 | 14.2 | 6172 | 2.3 |
| 21 | 6.8 | 8943 | 0.8 | 6.8 | 5437 | 1.3 |
| 22 | 4.1 | 4696 | 0.9 | 4.1 | 2341 | 1.8 |
| 23 | 5.7 | 6687 | 0.9 | 5.7 | 3780 | 1.5 |
| 24 | 6.3 | 7745 | 0.8 | 6.3 | 4686 | 1.3 |
| 25 | 2.2 | 2501 | 0.9 | 2.2 | 1315 | 1.7 |
| 26 | 5.3 | 6332 | 0.8 | 5.3 | 3289 | 1.6 |
| 27 | 5.2 | 5731 | 0.9 | 5.2 | 3254 | 1.6 |
| 28 | 4.7 | 5553 | 0.8 | 4.7 | 3240 | 1.5 |
| Z | 81.8 | 26642 | 3.1 | 0 | 0 | 0.0 |
| W | 0.9 | 14 | 64.3 | 0 | 0 | 0.0 |
| ^1^LGE22 | 1 | 213 | 4.7 | 0.9 | 119 | 7.6 |
| ^1^LGE64 | 0.8 | 89 | 9.0 | 0.6 | 36 | 16.7 |
| ^2^0 | 0 | 7883 | 0.0 | 0 | 4688 | 0.0 |
| Total | 1003.4 | 580961 | 1.7 | 919 | 348093 | 2.7 |

1 Linkage group, 2 these SNPs are not assigned to any chromosomes
