# Supplementary material for: Genome-wide association study and a post replication analysis revealed a promising genomic region and candidate genes for chicken eggshell blueness
Source: PLoS One. 2019 Jan 23;14(1):e0209181. doi: 10.1371/journal.pone.0209181 (PMC6343938; doi:10.1371/journal.pone.0209181)
Supplement: S2 Table — The values are given in (%). Met: methionine. (DOCX) [file pone.0209181.s002.docx]

**S2 Table.** Nutrition ingredients of the commercial diet for laying chickens

| Ingredient | Crude protein ≥ | Crude fat ≥ | Crude fiber ≤ | Crude ash ≤ | Calcium | Total phosphorus ≥ | NaCl | Met+Cystine ≥ | Water ≤ |
| --- | --- | --- | --- | --- | --- | --- | --- | --- | --- |
| Ration | 16.5 | 2.5 | 6 | 13 | 2.6-4 | 0.60 | 0.20-0.70 | 0.65 | 14.0 |

The values are given in (%). Met: methionine
