# Supplementary material for: Genome-wide association study and a post replication analysis revealed a promising genomic region and candidate genes for chicken eggshell blueness
Source: PLoS One. 2019 Jan 23;14(1):e0209181. doi: 10.1371/journal.pone.0209181 (PMC6343938; doi:10.1371/journal.pone.0209181)
Supplement: S3 Table — 1 GenBank Accession No. relates to the transcript used for the design of the primers. (DOCX) [file pone.0209181.s003.docx]

**S3 Table.** Primer pairs of the candidate genes used for quantitative real-time PCR

| **Serial** | | **Primer name** | **Primer sequences (5**′**- 3**′**)** | **Product size(bp)** | **T_a_(ºC)** | **GenBank Accession No.^1^** |
| --- | --- | --- | --- | --- | --- | --- |
| 1 | AJAP1-F | TCTTCACAGCCTACAACGAA | 202 | 59 | XM_015297071 |  |
|  | AJAP1-R | GCCAGTCAGCAGGAGATTT |  |  |  |  |
| 2 | CAMTA1-F | CAGCAGCACAAGCAAGGA | 133 | 58 | XM_417530 |  |
|  | CAMTA1-R | TCTTCTCGCACACCACCA |  |  |  |  |
| 3 | CEP104-F | GAAGTGGAGAAACGCTACGC | 146 | 58 | XM_004947360 |  |
|  | CEP104-R | GGCTTTCGAGACATCAGCTC |  |  |  |  |
| 4 | TNFRSF9-F | AATGGTGCCTGAGCCATAAC | 165 | 58 | XM_015297003 |  |
|  | TNFRSF9-R | TGAGCTGTGCTTTGAGAGGA |  |  |  |  |
| 5 | C1ORF174-F | TCTGCTGGCAAAACTCAATG | 130 | 59 | XM_417541 |  |
|  | C1ORF174-R | GGAAGATGGAAGCTGTGAGC |  |  |  |  |
| 6 | GAPDH-F | CTCTGTTGTTGACCTGACCT | 125 | 58-60 | NM_204305 |  |
|  | GAPDH-R | CAACCTGGTCCTCTGTGTAT |  |  |  |  |

1 GenBank Accession No. relates to the transcript used for the design of the primers
