# Supplementary material for: Genome-wide association study and a post replication analysis revealed a promising genomic region and candidate genes for chicken eggshell blueness
Source: PLoS One. 2019 Jan 23;14(1):e0209181. doi: 10.1371/journal.pone.0209181 (PMC6343938; doi:10.1371/journal.pone.0209181)
Supplement: S4 Table — (DOCX) [file pone.0209181.s004.docx]

**S4 Table.** Genomic inflation factors of the traits before and after inclusion PCA and MDS components as covariates in the linear squires regression model for GWAS

| Trait | GIF before inclusion PCA or MDS components as covariates in the model | GIF after inclusion leading MDS components as covariates in the model | GIF after inclusion leading PCA components as covariates in the model |
| --- | --- | --- | --- |
| **QB** | 2.02362 | 1.07847 (4 first components) | 1.04639 (16 first components) |
| **QP** | 3.05951 | 1.22964 (4 first components) | 1.08096 (14 first components) |
| **QT** | 2.58686 | 1.10444 (4 first components) | 1.04715 (16 first components) |
| **CD** | 2.29122 | 1.08837 (4 first components) | 1.00000 (17 first components) |
