# Supplementary material for: Genome-wide association study and a post replication analysis revealed a promising genomic region and candidate genes for chicken eggshell blueness
Source: PLoS One. 2019 Jan 23;14(1):e0209181. doi: 10.1371/journal.pone.0209181 (PMC6343938; doi:10.1371/journal.pone.0209181)
Supplement: S5 Table — 1Linkage group. 2These SNPs are not assigned to any chromosomes. (DOCX) [file pone.0209181.s005.docx]

**S5 Table.** Chromosome-wise significant threshold for each chromosome

| GGA | No. of SNP Markers | Indep. LD | LD blocks | Longest span (Kb) | Indep. LD + LD block | Significant threshold (Bonferroni correction) |
| --- | --- | --- | --- | --- | --- | --- |
| 1 | 62839 | 4890 | 11725 | 199.986 | 16615 | 6.019E-05 |
| 2 | 41969 | 3883 | 7928 | 199.967 | 11811 | 8.467E-05 |
| 3 | 36906 | 3385 | 7139 | 199.918 | 10524 | 9.502E-05 |
| 4 | 27587 | 2185 | 5354 | 199.992 | 7539 | 1.326E-04 |
| 5 | 20024 | 1852 | 3931 | 199.999 | 5783 | 1.729E-04 |
| 6 | 14660 | 1296 | 2838 | 199.719 | 4134 | 2.419E-04 |
| 7 | 14271 | 1387 | 2783 | 200 | 4170 | 2.398E-04 |
| 8 | 10887 | 935 | 2022 | 199.989 | 2957 | 3.382E-04 |
| 9 | 11906 | 1040 | 2351 | 197.007 | 3391 | 2.949E-04 |
| 10 | 11771 | 1036 | 2295 | 194.917 | 3331 | 3.002E-04 |
| 11 | 8232 | 680 | 1623 | 199.98 | 2303 | 4.342E-04 |
| 12 | 8769 | 724 | 1706 | 199.974 | 2430 | 4.115E-04 |
| 13 | 7125 | 725 | 1419 | 199.443 | 2144 | 4.664E-04 |
| 14 | 8657 | 837 | 1599 | 200 | 2436 | 4.105E-04 |
| 15 | 6520 | 583 | 1232 | 199.485 | 1815 | 5.510E-04 |
| 16 | 228 | 26 | 43 | 4.822 | 69 | 1.449E-02 |
| 17 | 5674 | 588 | 1107 | 196.332 | 1695 | 5.900E-04 |
| 18 | 6144 | 581 | 1120 | 196.84 | 1701 | 5.879E-04 |
| 19 | 5567 | 647 | 1043 | 198.055 | 1690 | 5.917E-04 |
| 20 | 6172 | 539 | 1277 | 199.97 | 1816 | 5.507E-04 |
| 21 | 5437 | 549 | 1096 | 155.608 | 1645 | 6.079E-04 |
| 22 | 2341 | 296 | 451 | 199.756 | 747 | 1.339E-03 |
| 23 | 3780 | 476 | 738 | 196.664 | 1214 | 8.237E-04 |
| 24 | 4686 | 536 | 894 | 104.276 | 1430 | 6.993E-04 |
| 25 | 1315 | 124 | 241 | 43.743 | 365 | 2.740E-03 |
| 26 | 3289 | 279 | 644 | 198.879 | 923 | 1.083E-03 |
| 27 | 3254 | 404 | 624 | 170.64 | 1028 | 9.728E-04 |
| 28 | 3240 | 369 | 622 | 197.273 | 991 | 1.009E-03 |
| Z | 1 | 1 | 0 | 0 | 1 | - |
| W | 0 | 0 | 0 | 0 | 0 | - |
| ^1^LGE22 | 119 | 27 | 18 | 31.348 | 45 | 2.222E-02 |
| ^1^LGE64 | 36 | 5 | 6 | 16.621 | 11 | 9.091E-02 |
| ^2^UN | 4687 | 0 | 604 | 0.001 | 604 | 1.656E-03 |
| Total | 348093 | 30885 | 66473 | 5101.204 | 97358 | 1.027E-05 |

^1^Linkage group.

^2^These SNPs are not assigned to any chromosomes.
