# Supplementary material for: Genome-wide association study and a post replication analysis revealed a promising genomic region and candidate genes for chicken eggshell blueness
Source: PLoS One. 2019 Jan 23;14(1):e0209181. doi: 10.1371/journal.pone.0209181 (PMC6343938; doi:10.1371/journal.pone.0209181)
Supplement: S6 Table — QB, QP, and QT (×10−8 mol/g). Values in bold indicate significant association with the trait. (DOCX) [file pone.0209181.s006.docx]

**S6 Table.** Additive and dominant effect of SNPs in N146 population on QB, QP, and QT traits

| **SNP ID** | **Genotype** | QB | QP | QT |
| --- | --- | --- | --- | --- |
| rs315477097 | Additive | 1.67±1.93 | 0.04±0.17 | 1.71±2.02 |
|  | P value | 0.3890 | 0.8036 | 0.3976 |
|  | Dominant | -2.92±2.35 | -0.05±0.21 | -2.97±2.45 |
|  | P value | 0.2155 | 0.8070 | 0.2276 |
| rs315586328 | Additive | -0.67±5.24 | 0.20±0.46 | -0.47±5.48 |
|  | P value | 0.8988 | 0.6660 | 0.9316 |
|  | Dominant | -2.79±5.61 | 0.50±0.49 | -2.29±5.87 |
|  | P value | 0.6198 | 0.3117 | 0.6962 |
| rs316706283 | Additive | -2.74±1.93 | -0.11±0.17 | -2.84±2.01 |
|  | P value | 0.1578 | 0.5363 | 0.1599 |
|  | Dominant | -4.52±2.35 | -0.17±0.21 | -4.68±2.45 |
|  | P value | 0.0561 | 0.4289 | 0.0579 |
| rs313867043 | Additive | 3.74±2.19 | 0.19±0.19 | 3.92±2.29 |
|  | P value | 0.0897 | 0.3315 | 0.0880 |
|  | Dominant | **-6.22±2.60** | -0.32±0.23 | **-6.55±2.72** |
|  | P value | **0.0181** | 0.1566 | **0.0173** |
| rs316919101 | Additive | -1.39±1.26 | -0.05±0.11 | -1.45±1.32 |
|  | P value | 0.2715 | 0.6122 | 0.2737 |
|  | Dominant | -2.83±1.72 | 0.11±0.15 | -2.71±1.80 |
|  | P value | 0.1021 | 0.4528 | 0.1332 |
| rs16177219 | Additive | 0.22±1.30 | 0.07±0.12 | 0.30±1.36 |
|  | P value | 0.8638 | 0.5409 | 0.8288 |
|  | Dominant | **-4.05±1.75** | -0.02±0.15 | **-4.07±1.82** |
|  | P value | **0.0217** | 0.8839 | **0.0270** |
| rs15180009 | Additive | -1.39±1.26 | -0.06±0.11 | -1.45±1.32 |
|  | P value | 0.2715 | 0.6122 | 0.2737 |
|  | Dominant | -2.83±1.72 | 0.11±0.15 | -2.71±1.80 |
|  | P value | 0.1021 | 0.4528 | 0.1332 |
| rs313199923 | Additive | 0.24±1.39 | -0.11±0.12 | 0.12±1.45 |
|  | P value | 0.8653 | 0.3365 | 0.9346 |
|  | Dominant | 0.96±1.84 | -0.04±0.16 | 0.91±1.92 |
|  | P value | 0.6051 | 0.7754 | 0.6373 |
| rs1617712 | Additive | -1.35±1.85 | -0.29±0.16 | -1.65±1.93 |
|  | P value | 0.4655 | 0.0711 | 0.3953 |
|  | Dominant | 0.82±2.25 | 0.20±0.19 | 1.02±2.35 |
|  | P value | 0.7162 | 0.3162 | 0.6658 |
| rs16177212 | Additive | -0.38±1.30 | -0.08±0.12 | -0.46±1.36 |
|  | P value | 0.7707 | 0.4976 | 0.7362 |
|  | Dominant | **-4.33±1.74** | -0.04±0.15 | **-4.36±1.82** |
|  | P value | **0.0140** | 0.8052 | **0.0176** |
| rs314071117 | Additive | 1.42±1.53 | 0.19±0.13 | 1.61±1.60 |
|  | P value | 0.3563 | 0.1531 | 0.3156 |
|  | Dominant | 1.77±2.01 | 0.17±0.17 | 1.94±2.09 |
|  | P value | 0.3804 | 0.3250 | 0.3561 |

QB, QP, and QT (×10^-8^ mol/g). Values in bold indicate signiﬁcant association with the trait.
