# Supplementary material for: Genome-wide association study and a post replication analysis revealed a promising genomic region and candidate genes for chicken eggshell blueness
Source: PLoS One. 2019 Jan 23;14(1):e0209181. doi: 10.1371/journal.pone.0209181 (PMC6343938; doi:10.1371/journal.pone.0209181)
Supplement: S7 Table — QB, QP, and QT (×10−8 mol/g). Values in bold indicate significant association with the trait. (DOCX) [file pone.0209181.s007.docx]

**S7 Table.** Additive and dominant effect of SNPs in N280 population on QB, QP, and QT traits

| **SNP ID** | **Genotype** | QB | QP | QT |
| --- | --- | --- | --- | --- |
| rs315477097 | Additive | 0.44±0.23 | -0.05±0.12 | 0.43±0.32 |
|  | P value | **0.0330** | 0.9686 | **0.0322** |
|  | Dominant | -0.47±0.33 | -0.35±0.17 | -0.81±0.47 |
|  | P value | 0.1617 | **0.0478** | 0.0820 |
| rs13602462 | Additive | -0.33±0.21 | 0.13±0.11 | -0.20±0.29 |
|  | P value | 0.1168 | 0.2476 | 0.4898 |
|  | Dominant | -0.06±0.33 | -0.02±0.17 | -0.09±0.47 |
|  | P value | 0.8457 | 0.8935 | 0.8501 |
| rs315586328 | Additive | 1.03±0.50 | -0.16±0.27 | 0.87±0.71 |
|  | P value | **0.0421** | 0.5562 | 0.2230 |
|  | Dominant | 0.01±0.70 | -0.01±0.37 | -0.00±0.99 |
|  | P value | 0.9857 | 0.9727 | 0.9998 |
| rs316706283 | Additive | -0.25±0.22 | 0.16±0.11 | -0.09±0.30 |
|  | P value | 0.2458 | 0.1613 | 0.7559 |
|  | Dominant | -0.12±0.35 | -0.17±0.18 | -0.29±0.49 |
|  | P value | 0.7308 | 0.3420 | 0.5491 |
| rs313867043 | Additive | 0.26±0.25 | -0.09±0.12 | 0.17±0.35 |
|  | P value | 0.3044 | 0.4851 | 0.6330 |
|  | Dominant | -0.20±0.34 | 0.00±0.17 | -0.18±0.47 |
|  | P value | 0.5651 | 0.9562 | 0.6959 |
| rs16177219 | Additive | 0.47±0.33 | -0.28±0.17 | 0.18±0.47 |
|  | P value | 0.1646 | 0.1054 | 0.6957 |
|  | Dominant | 0.30±0.49 | -0.39±0.26 | -0.09±0.69 |
|  | P value | 0.5415 | 0.1288 | 0.8968 |
| rs15180009 | Additive | -0.48±0.22 | 0.18±0.12 | -0.30±0.31 |
|  | P value | **0.0348** | 0.1166 | 0.3538 |
|  | Dominant | 0.44±0.33 | 0.13±0.17 | 0.57±0.46 |
|  | P value | 0.1881 | 0.4361 | 0.2195 |
| rs313199923 | Additive | -0.17±0.20 | -0.00±0.10 | -0.17±0.28 |
|  | P value | **0.0459** | 0.9991 | 0.2423 |
|  | Dominant | -0.40±0.31 | -0.09±0.16 | -0.49±0.43 |
|  | P value | 0.1924 | 0.5960 | 0.1575 |
| rs16177126 | Additive | -0.34±0.90 | 0.32±0.46 | -0.02±1.26 |
|  | P value | 0.7054 | 0.4866 | 0.9889 |
|  | Dominant | 0.23±0.99 | -0.83±0.51 | -0.61±1.38 |
|  | P value | 0.8180 | 0.1032 | 0.6604 |
| rs16177212 | Additive | -0.41±0.37 | 0.38±0.19 | -0.03±0.52 |
|  | P value | 0.2704 | **0.0487** | 0.9566 |
|  | Dominant | 0.17±0.46 | -0.32±0.24 | -0.15±0.65 |
|  | P value | 0.7050 | 0.1818 | 0.8213 |

QB, QP, and QT (×10^-8^ mol/g). Values in bold indicate signiﬁcant association with the trait.
