# Supplementary material for: Genome-wide association study and a post replication analysis revealed a promising genomic region and candidate genes for chicken eggshell blueness
Source: PLoS One. 2019 Jan 23;14(1):e0209181. doi: 10.1371/journal.pone.0209181 (PMC6343938; doi:10.1371/journal.pone.0209181)
Supplement: S8 Table — QB, QP, and QT (×10−8 mol/g). Means in the same column with different lowercase superscripts are different at P < 0.05. (DOCX) [file pone.0209181.s008.docx]

**S8 Table.** Associations of the haplotype combinations with eggshell color intensity traits in the N146 population of Dongxiang chicken (LSM±SE)

| Block ID | Haplotype combination  (No. individuals) | QB | QP | QT |
| --- | --- | --- | --- | --- |
| Block 1 | ACCGACCG (36) | 34.63± 1.73**^ab^** | 3.75±0.15 | 38.38±1.80**^ab^** |
|  | GCCGACTG (31) | 35.44±1.86**^ab^** | 4.01±0.16 | 39.44±1.94**^ab^** |
|  | GCCGAGTA (41) | 31.08±1.62**^ab^** | 3.72±0.14 | 34.80±1.69**^ab^** |
|  | GCCGAGTG (4) | 36.47±5.18**^ab^** | 4.68±0.45 | 41.15±5.41**^ab^** |
|  | GCTGGCTG (9) | 33.84±3.46**^ab^** | 3.64±0.30 | 37.49±3.61**^ab^** |
|  | GCTGGGTA (12) | 38.30±2.99**^ab^** | 3.87±0.26 | 42.17±3.13**^ab^** |
|  | GCTGGGTG (1) | 44.53±10.37**^a^** | 4.09±0.90 | 48.63±10.83**^a^** |
|  | GGTAGGTA (6) | 42.51±4.23**^a^** | 4.25±0.37 | 46.76±4.42**^a^** |
|  | GGTGGGTA (1) | 23.25±10.37**^b^** | 3.24±0.90 | 26.49±10.83**^b^** |
|  | P value | 0.1544 | 0.4609 | 0.1519 |
| Block 2 | CCCT (1) | 23.25±10.52 | 3.24±0.91 | 26.49±11.01 |
|  | CTCT (7) | 39.51±4.30 | 4.06±0.37 | 43.58±4.49 |
|  | GCCT (49) | 34.66±1.52 | 3.81±0.13 | 38.47±1.59 |
|  | GCGC (50) | 34.13±1.50 | 3.74±0.13 | 37.87±1.57 |
|  | GCGT (25) | 35.89±2.10 | 4.01±0.18 | 39.90±2.20 |
|  | GTCT (11) | 28.74±3.33 | 4.15±0.29 | 32.89±3.48 |
|  | GTGT (2) | 35.63±7.44 | 3.58±0.64 | 39.21±7.78 |
|  | P value | 0.4258 | 0.7325 | 0.4769 |
| Block 3 | AAGGGT (5) | 33.09±1.44 | 3.73±0.12 | 36.82±1.50 |
|  | AATAAT (9) | 32.08±3.45 | 3.35±0.30 | 35.43±3.61 |
|  | AATGAT (13) | 39.30±2.87 | 4.12±0.25 | 43.42±3.00 |
|  | GAGGGG (1) | 32.25±10.35 | 3.24±0.90 | 26.49±10.82 |
|  | GAGGGT (24) | 31.09±2.16 | 4.00±0.19 | 35.09±2.26 |
|  | GATGAT (2) | 35.63±7.32 | 3.58±0.64 | 39.21±7.65 |
|  | GGGGGG (43) | 37.14±1.62 | 3.96±0.14 | 41.10±1.69 |
|  | P value | 0.1107 | 0.3634 | 0.1147 |

QB, QP, and QT (×10^-8^ mol/g). Means in the same column with different lowercase superscripts are different at P<0.05.
