# Supplementary material for: Genome-wide association study and a post replication analysis revealed a promising genomic region and candidate genes for chicken eggshell blueness
Source: PLoS One. 2019 Jan 23;14(1):e0209181. doi: 10.1371/journal.pone.0209181 (PMC6343938; doi:10.1371/journal.pone.0209181)
Supplement: S9 Table — QB, QP, and QT (×10−8 mol/g). Means in the same column with different lowercase superscripts are different at P < 0.05. Values in bold indicate significant association with the trait. (DOCX) [file pone.0209181.s009.docx]

**S9 Table.** Associations of the haplotype combinations with eggshell color intensity traits in the N280 population of Dongxiang chicken (LSM±SE).

| Block ID | Haplotype combination  (No. individuals) | QB | QP | QT |
| --- | --- | --- | --- | --- |
| Block 1 | CCCC (38) | 10.26±0.41**^a^** | 4.30±0.21 | 14.56±0.58 |
|  | CCCT (65) | 11.32±0.31**^ab^** | 4.41±0.16 | 15.73±0.44 |
|  | CCGT (52) | 11.00±0.35**^ab^** | 3.97±0.18 | 14.97±0.49 |
|  | CTCT (33) | 10.98±0.44**^ab^** | 3.82±0.23 | 14.80±0.62 |
|  | CTGT (62) | 11.22±0.32**^ab^** | 3.84±0.17 | 15.06±0.45 |
|  | GTGT (30) | 11.53±0.46**^b^** | 3.92±0.24 | 15.45±0.65 |
|  | P value | 0.3334 | 0.1060 | 0.6272 |
| Block 2 | CCCT (4) | 13.00±1.26**^a^** | 5.74±0.65**^a^** | 18.74±1.76**^a^** |
|  | CTCT (33) | 11.71±0.44**^ab^** | 3.95±0.23**^b^** | 15.66±0.61**^ab^** |
|  | GCCC (4) | 8.99±1.26**^c^** | 4.08±0.65**^b^** | 13.08±1.76**^b^** |
|  | GCCT (102) | 10.91±0.25**^abc^** | 3.81±0.13**^b^** | 14.72±0.35**^b^** |
|  | GCGC (82) | 11.19±0.28**^abc^** | 4.10±0.14**^b^** | 15.29±0.39**^ab^** |
|  | GCGT (40) | 10.73±0.40**^abc^** | 4.39±0.21**^b^** | 15.12±0.56**^b^** |
|  | GTCT (7) | 12.59±0.95**^ab^** | 4.15±0.49**^b^** | 16.74±1.33**^ab^** |
|  | GTGT (8) | 10.01±0.89**^bc^** | 4.79±0.46**^ab^** | 14.80±1.25**^b^** |
|  | P value | 0.0897 | **0.0295** | 0.2313 |
| Block 3 | ACAGGCGT (15) | 11.50±0.65**^ab^** | 3.64±0.33**^a^** | 15.15±0.92**^ab^** |
|  | ACAGGTGT (23) | 10.66±0.53**^ab^** | 3.54±0.27**^a^** | 14.20±0.74**^a^** |
|  | ACATACAT (2) | 10.39±1.79**^ab^** | 4.68±0.92**^ab^** | 15.07±2.51**^ab^** |
|  | ATGGGTGG (1) | 10.27±2.54**^ab^** | 3.26±1. 30**^a^** | 13.53±3.55**^a^** |
|  | GCAGGCAT (2) | 11.48±1.79**^ab^** | 4.93±0.92**^ab^** | 16.40±2.51**^ab^** |
|  | GCAGGCGG (4) | 9.00±1.27**^a^** | 4.08±0.65**^a^** | 13.08±1.78**^a^** |
|  | GCAGGCGT (32) | 10.53±0.45**^ab^** | 4.43±0.23**^a^** | 14. 96±0.63**^ab^** |
|  | GCAGGTGG (3) | 13.67±1.46**^b^** | 6.45±0.75**^b^** | 20.12±2.05**^b^** |
|  | GCAGGTGT (14) | 11.67±0.68**^ab^** | 4.13±0.35**^a^** | 15.80±0.95**^ab^** |
|  | GCATGCAT (8) | 10.01±0.90**^ab^** | 4.79±0.46**^ab^** | 14.80±1.26**^ab^** |
|  | GCGGGCGG (58) | 11.09±0.33**^ab^** | 4.04±0.17**^a^** | 15.13±0.47**^ab^** |
|  | GCGGGTGG (83) | 11.11±0.28**^ab^** | 4.03±0.14**^a^** | 15.15±0.39**^ab^** |
|  | GTGGGTGG (34) | 11.63±0.43**^ab^** | 3.91±0.22**^a^** | 15.54±0.61**^ab^** |
|  | P value | 0.4149 | **0.0355** | 0.5822 |

QB, QP, and QT (×10^-8^ mol/g). Means in the same column with different lowercase superscripts are different at P<0.05. Values in bold indicate signiﬁcant association with the trait.
